# Gastrotomy approach for removal of an oesophageal foreign body in a dog

**DOI:** 10.1002/vms3.1098

**Published:** 2023-03-03

**Authors:** Tomohiro Osaki, Yusuke Murahata, Aiko Iguchi, Takao Amaha, Yoshiharu Okamoto

**Affiliations:** ^1^ Joint Department of Veterinary Clinical Medicine Faculty of Agriculture, Tottori University Tottori Japan

**Keywords:** dog, foreign body, gastrotomy, long forceps, oesophagus, radiographic

## Abstract

A 9‐year‐old castrated male Kaninchen dachshund dog weighing 4.18 kg was referred to our institution and presented with occasional vomiting and dysphagia. The radiographic examination revealed a long radiopaque foreign body located throughout the thoracic oesophagus. Endoscopic removal was attempted using laparoscopic forceps but was unsuccessful as the foreign body was too large to be grasped. A gastrotomy was therefore performed, and long paean forceps were gently and blindly inserted into the cardia of the stomach. The bone foreign body was grasped with the long paean forceps under fluoroscopy and withdrawn from the oesophagus while checking with an endoscope. A gastrotomy approach using long forceps, endoscopy and fluoroscopy should be considered for removal of oesophageal foreign bodies from patients in which an endoscopic approach has been unsuccessful.

## Graphical abstract

The radiographic examination in Kaninchen dachshund dog revealed a long radiopaque foreign body located throughout the thoracic oesophagus. A gastrotomy was performed. The bone foreign body was grasped with the long paean forceps under fluoroscopy and withdrawn from the oesophagus while checking with an endoscope.

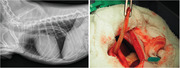

## 1 INTRODUCTION

Oesophageal foreign bodies (EFBs) are inanimate objects that do not normally advance into the stomach after being ingested. EFBs are commonly found immediately caudal to the pharynx, thoracic inlet, heart base and distal to the oesophagus. These objects might induce oesophageal obstruction to varying degrees (Radlinsky & Fossum, 2019; Runge & Culp, 2013). The most common foreign body type in dogs is ingested bones or bone fragments (Kyles & Huck, 2018). The usual treatment for EFBs is endoscopic removal or, if this is not possible, advancement of the foreign body into the stomach can be attempted (Runge & Culp, 2013). If neither technique is successful to facilitate anterograde removal, the use of long forceps might be performed (Yoon et al., 2009). However, removal of a long foreign body using this technique has not previously been described. This case report describes gastrotomy for retrieval of a long foreign body that occupied the full length of the thoracic oesophagus.

## 2 CASE PRESENTATION

**FIGURE 1:**
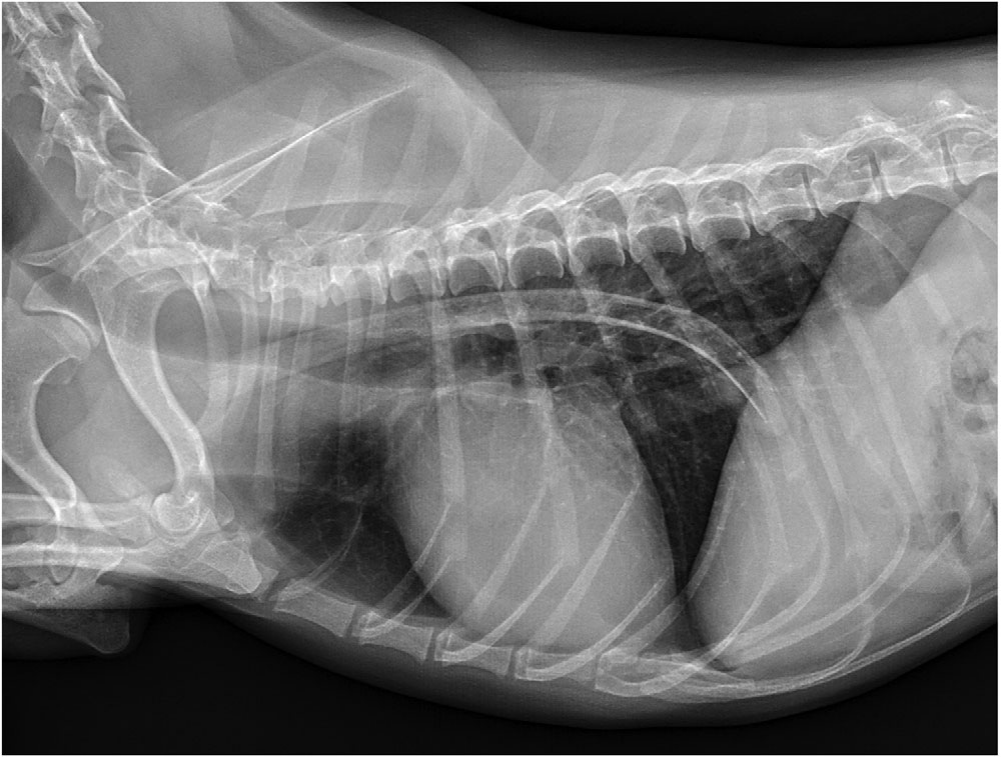
Right lateral thoracic radiography showing a radiodense oesophageal foreign body.

A 9‐year‐old castrated male Kaninchen dachshund, weighing 4.18 kg, was referred to our institution. He presented with occasional vomiting and dysphagia. According to the owner, the dog had picked through the garbage a week earlier. Radiographic examination revealed a long, radiopaque foreign body in the thoracic oesophagus (Figure 1). Radiographic images showed no abnormal findings other than a foreign body. The foreign body was identified as a lamb bone. Complete blood count and routine serum biochemistry were normal. Removal of the foreign body was performed at the first visit.

**FIGURE 2:**
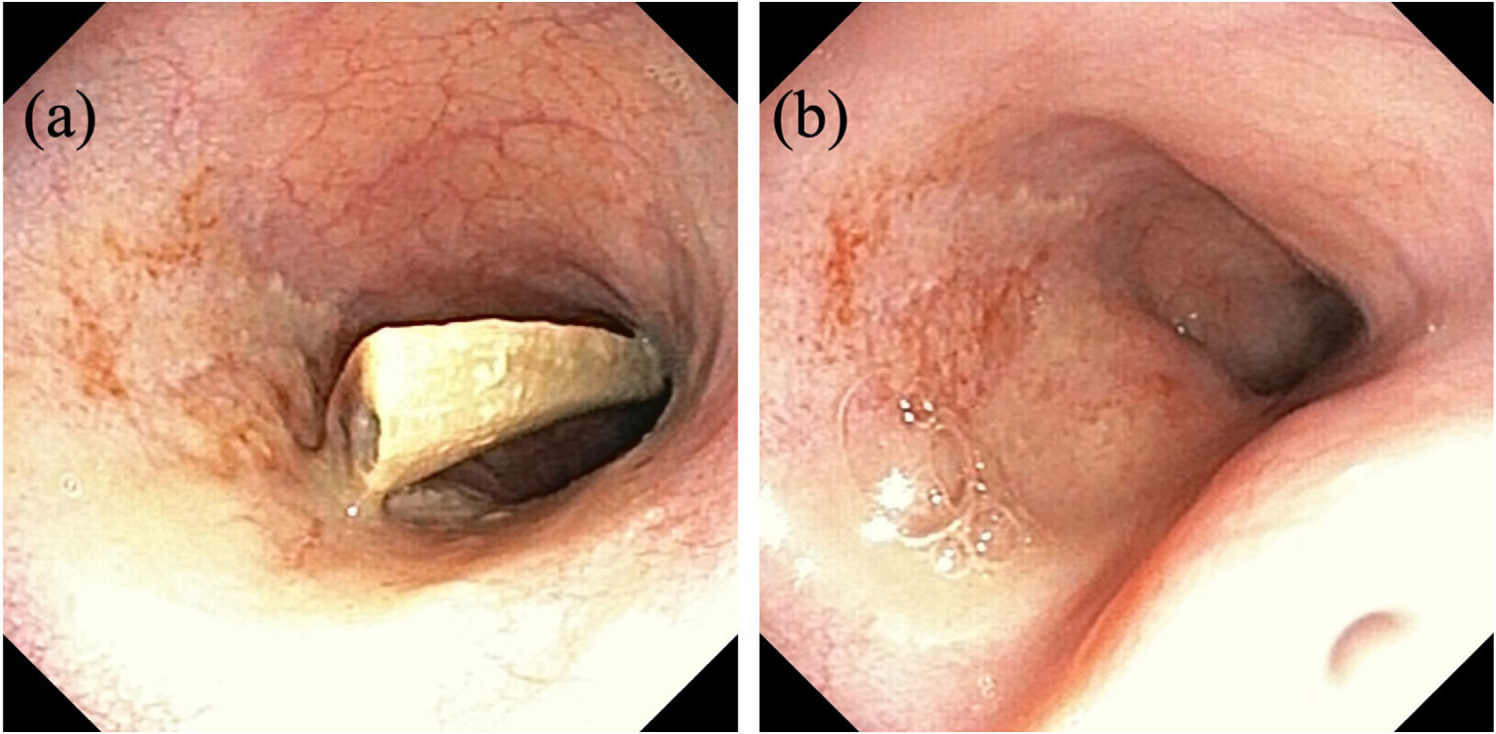
Endoscopic image showing proximal aspect of the oesophageal foreign body. (a) Before the removal of the foreign body. (b) After the removal of the foreign body.

**FIGURE 3:**
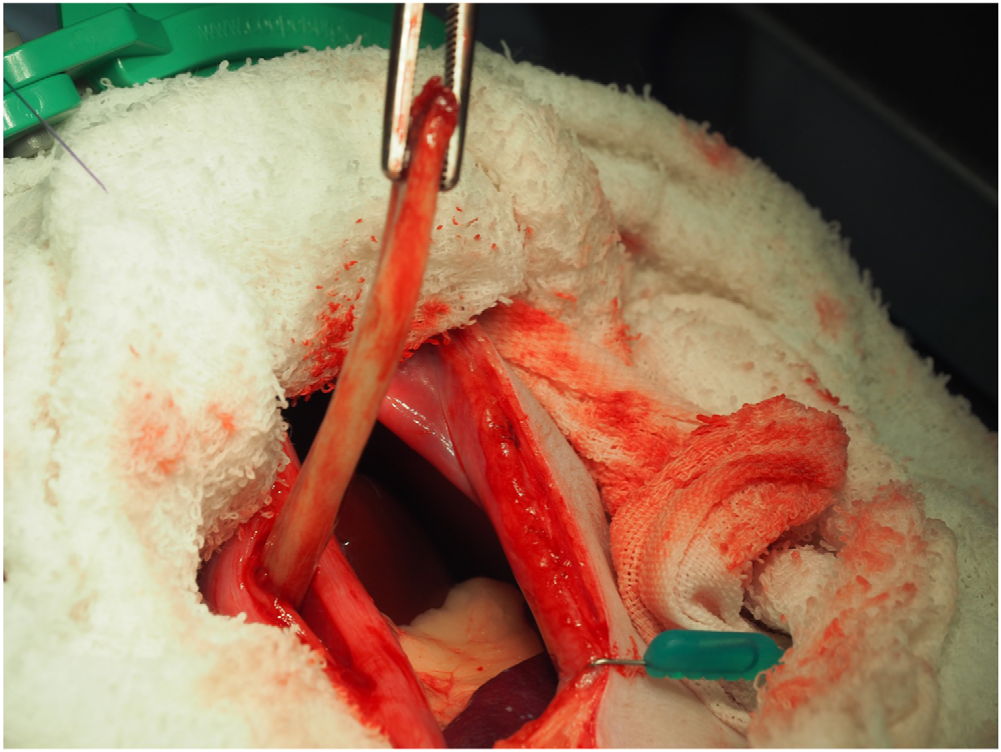
Intraoperative image showing withdrawal of oesophageal foreign body from the cardia using paean forceps.

**FIGURE 4:**
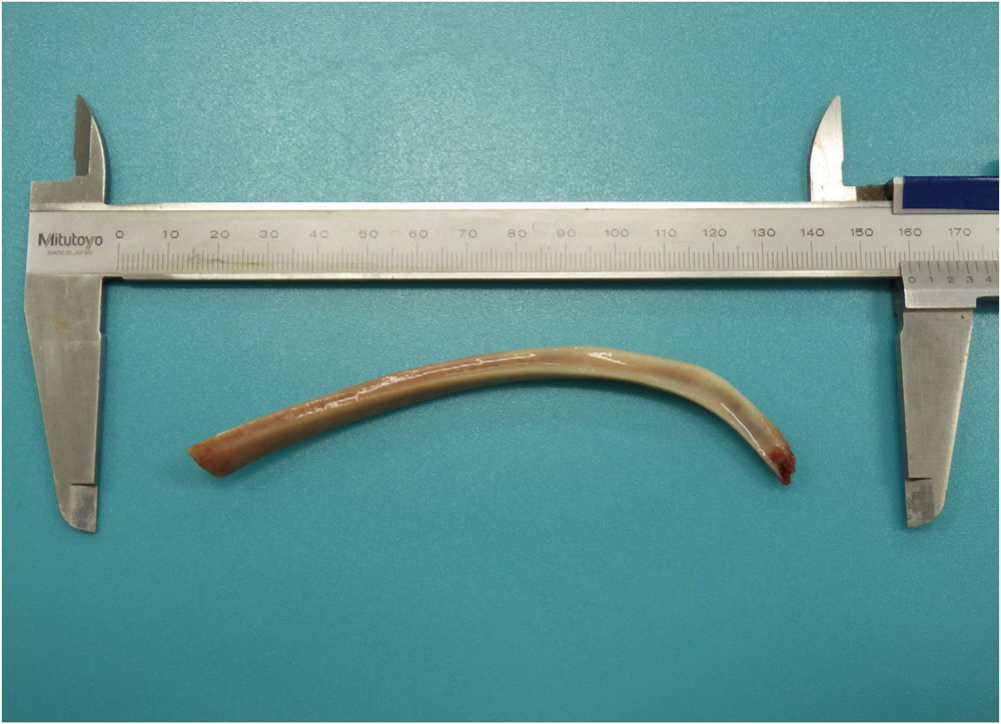
Extracted foreign bone.

The dog was premedicated with atropine sulphate (0.015 mg/kg IV; Atropine Sulphate Injection 0.5 mg, Nipro ES Pharma, Osaka, Japan) and fentanyl citrate (5 μg/kg IV; Fentanyl Injection 0.25 mg, Terumo, Tokyo, Japan). General anaesthesia was induced with intravenous propofol (4 mg/kg; PropoFlo 28, Zoetis Japan, Tokyo, Japan). After tracheal intubation, anaesthesia was maintained using end‐tidal isoflurane (Isoflu; DS Pharma Animal Health, Osaka, Japan) at 0.8%–1.2% and oxygen with mechanical ventilation. Intraoperative analgesia was provided using fentanil citrate at 15 μg/kg/h constant rate infusion and robenacoxib (2 mg/kg SC; Onsior, Elanco Japan, Tokyo, Japan). Removal of the foreign body was initially attempted endoscopically using laparoscopic forceps (Figure 2); however, the foreign body was too large. Therefore, laparotomy was performed. A ventral midline incision was made from the xiphoid process to the umbilicus. Then, the stomach was isolated from the surrounding organs with a moistened surgical towel. An incision was made between two stay sutures, whereas gastric contents were suctioned using an aspirator. Long paean forceps were blindly inserted into the cardia of the stomach. Finally, the foreign bone was grasped with the forceps under fluoroscopy. The curved part of the bone did not easily pass through the cardia when gentle traction was applied; however, after guiding the cardia to the tip of the bone with tweezers, the bone was successfully withdrawn from the oesophagus under endoscopic guidance (Figure 3). Oesophageal endoscopy revealed moderate trauma to the oesophageal mucosa but with no evidence of laceration or penetration. The bone measured 120 mm in length (Figure 4). The stomach was sutured in two layers using 3‐0 polydioxanone (PDS, Ethicon Inc., Johnson & Johnson Medical Ltd., Tokyo, Japan). The linea alba and subcutaneous tissue were sutured using 3‐0 polydioxanone. The skin was closed using a skin stapler (Visistat35W, Teleflex Medical Japan, Tokyo, Japan). Postoperative analgesia was provided using fentanil citrate at 3 μg/kg/h CRI for the initial 3 h, and thereafter with buprenorphine hydrochloride (Lepetan injection 0.2 mg; Otsuka Pharmaceutical) at 20 μg/kg IV q8h. Perioperatively, famotidine (1 mg/kg; Gaster injection 10 mg, LTL pharma, Tokyo, Japan) and sucralfate hydrate (Ulcerlmin, Fuji Chemical Industries, Toyama, Japan) were used for mucosal protection. The dog made an uneventful recovery and was discharged the next day. When visiting the referral hospital 67 days post‐procedure, there were no ongoing gastrointestinal signs or other clinical concerns.

## 3 DISCUSSION

As in the present case, most EFBs are radiopaque and easily detected by radiography. Bones are the most common cause of oesophageal obstruction ranging in incidence from 29.7% to 80% (Luthi & Neiger, 1998; Rousseau et al., 2007; Thompson et al., 2012). The owner identified the foreign body as a rib bone that they had previously thrown away. Bony EFBs are commonly found at the distal thoracic oesophagus, followed by the heart base, caudal pharynx and thoracic inlet (Thompson et al., 2012). There were few reports of foreign bodies present throughout the thoracic oesophagus.

Retrieval of ESBs is usually attempted endoscopically using grasping forceps or a balloon catheter. Success rates of endoscopic removal or dislodgement have been reported as 95%, and dislodgement was impossible in three dogs with moderate‐to‐severe oesophagitis induced by ESBs (Rousseau et al., 2007). In the present case, endoscopic removal was unsuccessful as the foreign body was too large to be grasped with the forceps. A gastrotomy approach was, therefore, performed using a long forceps technique under fluoroscopy. The foreign body did not easily pass through the cardia due to its curved shape; however, successful removal was achieved under fluoroscopy by guiding the cardia to the tip of the bone with tweezers which enabled subsequent withdrawal from the oesophagus. In a previous report, EFBs were blindly palpated through the oesophageal hiatus and retrieved by gastrotomy using the long forceps technique (Yoon et al., 2009). The authors did not use an endoscope during the surgery and thus were unable to assess the oesophageal mucosa. In this present study, the foreign body was easily grasped under fluoroscopy and concurrent endoscopic visualisation facilitated assessment of any oesophageal mucosal damage. Therefore, sucralfate was used only perioperatively. As no surgical complications were observed in the present case, this technique could help avoid the need for more invasive oesophageal surgery. However, complications such as iatrogenic damage or perforation caused by forceps must be considered. Delayed complication, such as stricture, occurring a few weeks after EFB removal, may also result from damage to the deeper layers of the oesophageal wall (Rousseau et al., 2007; Kyles & Huck, 2018; Radlinsky & Fossum, 2019).

## 4 CONCLUSION

A foreign body that occupied the full length of the thoracic oesophagus was successfully removed using a gastrotomy approach aided by fluoroscopy and endoscopy. A good outcome was achieved with no complications. This technique should be considered for patients in which an endoscopic approach has been unsuccessful.

## AUTHOR CONTRIBUTIONS

*Conceptualization‐lead; data curation‐lead; formal analysis‐lead; funding acquisition‐lead; investigation‐lead; methodology‐lead; project administration‐lead; supervision‐lead; validation‐lead; visualization‐lead; writing – original draft preparation‐lead; writing – review and editing‐lead*: Tomohiro Osaki. *Data curation‐supporting; investigation‐supporting; visualization‐supporting; writing – review and editing‐supporting*: Yusuke Murahata. *Data curation‐supporting; investigation‐supporting; writing – review and editing –supporting*: Aiko Iguchi. *Data curation‐supporting; investigation‐supporting; visualization‐supporting; writing – review and editing – supporting*: Takao Amaha. *Supervision‐supporting; writing – review and editing – equal*: Yoshiharu Okamoto.

## CONFLICT OF INTEREST STATEMENT

The authors declared no conflicts of interests.

### ETHICS STATEMENT

Approval from the Ethics Committee was not needed for the completion of this case report.

### PEER REVIEW

The peer review history for this article is available at https://publons.com/publon/10.1002/vms3.1098.

## FUNDING

This research received no specific grant from any funding agency in the public, commercial, or not‐for‐profit sectors.

## Data Availability

The data that support the findings of this study are available from the corresponding author upon reasonable request.
